# Postkala-Azar Dermal and Visceral Leishmaniasis in a Patient from Bangladesh

**DOI:** 10.4269/ajtmh.26-0159

**Published:** 2026-05-21

**Authors:** Rowena Lee Ying Kwan, Hui Yi Chia, Shawn Vasoo, Jiun Yit Pan

**Affiliations:** ^1^ Yong Loo Lin School of Medicine, National University of Singapore, Singapore;; ^2^ National Skin Centre, Singapore;; ^3^ National Centre for Infectious Diseases, Singapore

**Figure 1.**
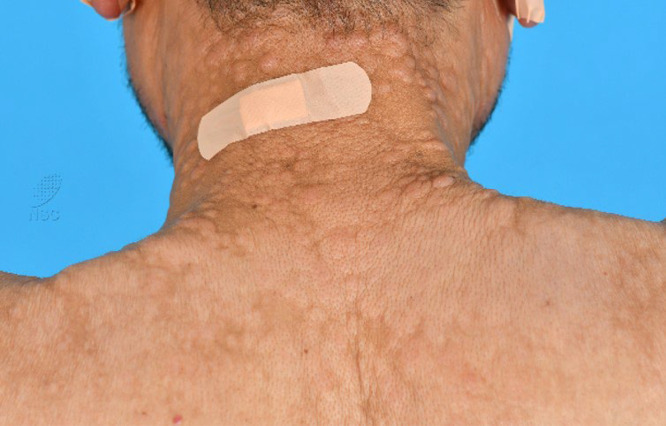
Infiltrated hypopigmented plaques on the nape of the neck and upper back.

A 35-year-old Bangladeshi man presented in April 2024 with a 1-year history of infiltrated plaques and hypopigmented patches on the face, ears, neck, and trunk (Figure 1). Slit-skin smears (SSSs) from both earlobes and the right elbow were positive for acid-fast bacilli. Chest histology showed superficial perivascular chronic inflammation without granulomas and was negative for acid-fast bacilli on Ziehl–Neelsen and Fite stains. He was diagnosed with leprosy in reaction and treated with WHO multidrug therapy (dapsone, rifampicin, and clofazimine) and prednisolone. His lesions initially resolved. However, tapering prednisolone from August 2024 triggered repeated reactional states with fevers, skin lesion flares, and blurred vision.

**Figure 2.**
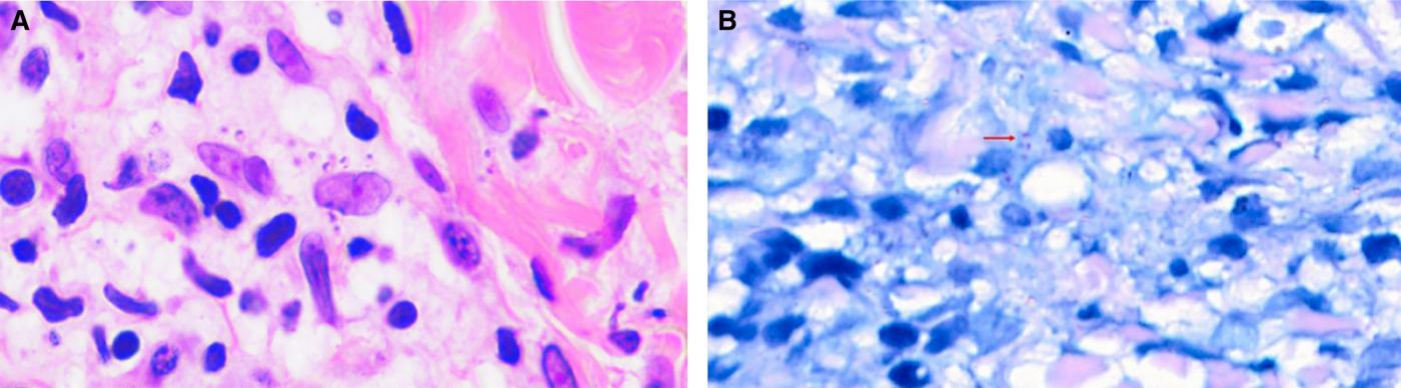
Some spherical structures measuring 1 *µ*m were seen within histiocytes (hematoxylin and eosin, 1,000× magnification; left panel). These organisms stained positively on Giemsa stain (right panel).

The patient was readmitted in February 2025 for intermittent fevers, hepatosplenomegaly, and bilateral small-volume axillary and inguinal lymphadenopathy. Upon questioning, he recalled treatment of visceral leishmaniasis (VL; kala-azar) more than 15 years earlier. Investigations were negative for leptospirosis, malaria, influenza, dengue, HIV, Zika, chikungunya, and acute Epstein–Barr virus infection. Blood and urine cultures were negative. Given the discordant clinical progression and poor therapeutic response, his skin biopsy was re-evaluated with additional stains (Figure 2), revealing multiple *Leishmania* amastigotes on Giemsa stain.

Considering his prior infection, biopsy findings, and systemic features, his presentation was consistent with parakala-azar dermal leishmaniasis (para-KDL), which is defined as concurrent postkala-azar dermal leishmaniasis (PKDL) and VL.^1^ This likely represented a relapse or incompletely treated previous VL. His cutaneous lesions represented PKDL mimicking leprosy, whereas SSS positivity was attributed to staining artefacts or crosscontamination. His initial improvement under leprosy therapy likely reflected corticosteroid-mediated anti-inflammatory effects masking his symptoms, whereas their immunosuppressive action may have contributed to development of para-KDL.

The patient self-discharged to return to Bangladesh, where he was informed of his diagnosis and advised to seek local follow-up.

Postkala-azar dermal leishmaniasis is a cutaneous sequela of VL that is caused by *Leishmania* parasites and transmitted by *Phlebotomus* sandflies, arising in patients with or without prior visceral disease.^2^^,^^3^ Postkala-azar dermal leishmaniasis is predominantly associated with *Leishmania donovani*, and it is mostly found on the Indian subcontinent and less commonly, in Africa.^3^ Parakala-azar dermal leishmaniasis affects approximately 1 in 700 PKDL patients on the Indian subcontinent.^4^ Treatment of PKDL and para-KDL includes miltefosine and liposomal amphotericin B.^1^

Postkala-azar dermal leishmaniasis may mimic leprosy and should be considered in leprosy-suspect patients from VL-endemic regions. Therefore, excluding PKDL with appropriate testing, including molecular methods, is essential before commencing leprosy treatment. Because these dermal forms serve as reservoirs of infection,^1^^–^^3^ prompt recognition is essential to successfully eliminate VL.
